# Driving proteomic imbalance in malignancy provokes proteomic catastrophe and confers tumor suppression

**DOI:** 10.1101/2024.05.24.595838

**Published:** 2026-03-06

**Authors:** Babul Moni Ram, Omprakash Shriwas, Meng Xu, Kun-Han Chuang, Chengkai Dai

**Affiliations:** 1 Mouse Cancer Genetics Program, Center for Cancer Research, National Cancer Institute, Frederick, MD 21702, USA; 2 Present address: School of Basic Medical Sciences, Fujian Medical University, Fuzhou, Fujian 350108, China

## Abstract

Unlike genomic instability, the implications of proteomic instability in cancer remain ambiguous. By governing the proteotoxic stress response, heat shock factor 1 (HSF1) sustains proteomic stability upon environmental insults. Apart from its importance to stress resistance and survival, HSF1 is emerging as a powerful oncogenic enabler. In the Neurofibromatosis type I (*NF1*)-deficient malignant peripheral nerve sheath tumor (MPNST) cells, *HSF1* depletion triggered protein polyubiquitination, aggregation, and even tumor-suppressive amyloidogenesis. In contrast, *HSF1* is dispensable for the proteome of non-transformed human Schwann cells. Mechanistically, HSF1 defends the essential mitochondrial chaperone HSP60 against the direct assault from soluble amyloid oligomers. To survive and adapt to compromised protein quality, owing to *HSF1* deficiency, MPNST cells mobilized JNK to repress mTORC1 and protein translation, thereby attenuating protein quantity to alleviate proteomic imbalance. mTORC1 stimulation, via either pharmacological JNK blockade, genetic *TSC2* depletion, or leucine supplementation, markedly aggravated the proteomic imbalance elicited by *HSF1* deficiency. This catastrophic imbalance instigated pronounced cell death partly through unchecked amyloidogenesis, thereby exerting tumor suppression in both MPNST and melanoma models *in vivo*. Thus, HSF1 safeguards the cancer proteome to enable the oncogenic potential of mTORC1. This proof-of-principle study highlights provoking proteomic catastrophe as a next-generation therapeutic concept for combating malignancy.

## INTRODUCTION

Compared to genomic instability, proteomic instability in cancer is underappreciated. Malignant transformation prompts proteotoxic stress and destabilizes the proteome.^1, 2^ Several mechanisms, including reactive oxygen species, genetic mutations, and uncontrolled protein translation, all lead to increased protein damage, misfolding, as well as aggregation.^1–3^ Of great interest, amyloidogenesis, a hallmark of neurodegenerative disorders,^4^ also occurs in cancer.^5^ Unlike neurons, cancerous cells manage to contain amyloidogenesis and counteract the cytotoxic effects of amyloids. Among various cellular anti-amyloid mechanisms is heat shock factor 1 (HSF1) and its mediated heat shock response or proteotoxic stress response (HSR/PSR), a powerful cytoprotective transcriptional program.^6–8^ In accordance with its role in repressing proteomic instability, HSF1 has emerged as a potent oncogenic enabler, independently of driver mutations.^9–15^

Sharply contrasting with its dispensability for primary and non-transformed cells, HSF1 becomes essential to numerous cancer cell lines,^9,13,16–18^ owing to their elevated intrinsic proteotoxic stress. This phenomenon is referred to as “HSF1 or non-oncogene addiction of cancer”,^2,19^ justifying HSF1 as a promising therapeutic target.

Neurofibromatosis type 1 (NF1) is a cancer predisposition syndrome afflicting approximately 1 in 3000 individuals worldwide.^20^ About 30–50% of NF1 individuals develop benign plexiform neurofibromas, which, unlike dermal neurofibromas, can transform into highly aggressive malignant peripheral nerve sheath tumors (MPNSTs) with poor prognosis and survival.^21^ The *NF1* gene encodes neurofibromin, a 250–280 kDa RAS GTPase activating protein (GAP) that negatively regulates RAS activity.^22^ *NF1* inactivation in MPNST cells dysregulates RAS/MAPK signaling which, in addition to stimulating cell growth, leads to constitutive HSF1 activation.^13^ Concomitantly, HSF1 overexpression and activation are seen in both *in vitro* and *in vivo* MPNST models as well as primary NF1 tumors.^13^ Importantly, *Hsf1* deficiency impaired the NF1-associated tumorigenesis in mice in part by attenuating oncogenic RAS signaling.^13^

Utilizing the NF1 tumor model system, we herein demonstrate HSF1 as a key player in countering proteomic instability in cancer. We discover that amyloidogenesis occurs in *NF1-*deficient MPNST cells, partly due to hyperactive mTORC1 and augmented protein translation. HSF1 inhibition destabilizes the cancer proteome, promoting global protein polyubiquitination, aggregation, as well as amyloidogenesis. Importantly, this proteomic instability causes cytotoxicity. To adapt and survive HSF1 inhibition, MPNST cells mobilize JNK to suppress mTORC1 and thereby mitigate protein translation. Disrupting this adaptative response through JNK inhibition aggravates amyloidogenesis and cytotoxicity. Similarly, mTORC1 stimulation, via either genetic *TSC2* depletion or supplementation with leucine or its analog, synergizes with HSF1 inhibition to inflict profound proteomic chaos and widespread non-apoptotic cell death. Of note, blockade of amyloidogenesis markedly alleviates cell death. Thus, our findings illuminate a prominent role of HSF1 in antagonizing proteomic instability in cancerous cells, constituting a generic pro-oncogenic mechanism. This study exemplifies a proof of principle and, importantly, institutes provoking proteomic catastrophe as a next-generation anti-cancer therapeutic concept.

## RESULTS

### HSF1 counters proteomic instability in *NF1*-deficient MPNST cells

**Figure 1:**
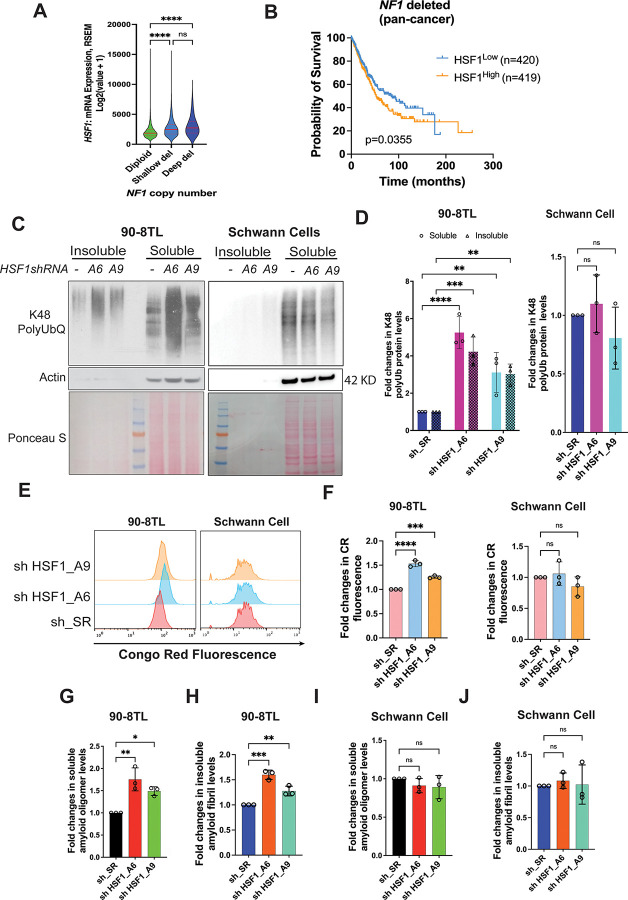
HSF1 is required to suppress proteomic instability and amyloidogenesis in MPNST cells. (A) Violin plots of *HSF1* expression in human cancer tissues showing *NF1* deletion (median and IQR, n=6424, 1598, or 88, Kruskal-Wallis test). Data are generated by the TGCA Research Network (https://www.cancer.gov/tcga). (B) Kaplan-Meier survival curves of patients whose tumors harbor *NF1* deletion (n=420 or 419, Log-rank test). Data are generated by the TGCA Research Network (https://www.cancer.gov/tcga). (C) and (D) The impacts of *HSF1* depletion on protein polyubiquitination in both 90-8TL cells and primary human Schwann cells. Cells were transduced with lentiviral shRNAs for 4 days and global protein Lys48-specific ubiquitination was detected by immunoblotting in both detergent-soluble and -insoluble fractions of whole cell lysates (C). Protein ubiquitination was quantitated using Fiji imaging software (D) (mean ± SD, n=3 independent experiments, Two-way ANOVA). (E) and (F) Congo Red (CR) staining of 90-8TL cells and primary human Schwann cells with and without *HSF1* depletion, analyzed by flow cytometry (E). Quantitation of CR staining using the median fluorescence intensity (F) (mean ± SD, n=3 independent experiments, One-way ANOVA). (G)-(J) Quantitation of soluble amyloid oligomers and insoluble amyloid fibrils by ELISA using A11 and OC antibodies, respectively (mean ± SD, n=3 independent experiments, One-way ANOVA).

Previously, we found that loss of *NF1* leads to constitutive activation of HSF1 and that *Hsf1* deficiency impairs the tumorigenesis in *Nf1*^+/−^; *p53*^+/−^ (NPcis) mice.^13^ In human cancers, *NF1* deletion is associated with elevated *HSF1* gene expression (Figure 1A). Moreover, the expression of *NF1* and *HSF1* genes are inversely correlated in human cancers (Figure S1A). Importantly, among patients whose tumors harbor *NF1* deletion higher *HSF1* expression is correlated with shortened survival (Figure 1B). Collectively, these findings support a pro-oncogenic role of HSF1 in *NF1*-deficient malignancies.

Given its critical role in governing protein folding, we were curious about how *HSF1* depletion impacts the proteomes of *NF1*-deficient MPNST cells as well as *NF1*-proficient non-transformed human Schwann cells (HSCs), both primary and immortalized. HSCs are considered as the cell-of-origin of MPNSTs.^20,22^ In three different MPNST cell lines (90-8TL, S462, and SNF96.2), transient depletion of *HSF1*, using two previously validated lentiviral shRNAs,^9,13^ all caused an increase in global protein Lys48-specific polyubiquitination, a molecular mark for proteasomal degradation,^23^ in both detergent-soluble and -insoluble fractions (Figures 1C, 1D, S1B, and S1C). This increased polyubiquitination signifies proteome-wide misfolding and aggregation. In stark contrast, this change was not observed in HSCs (Figures 1C and 1D). Thus, *HSF1* depletion provokes proteomic instability in MPNST cells, but not in HSCs.

Most cellular protein aggregates are amorphous; however, a tiny fraction becomes highly ordered and enriched with β-sheet structures. These special protein aggregates, namely amyloids, are closely associated with neurodegenerative disorders in humans, especially Alzheimer’s disease.^24^ To assess whether *HSF1* depletion could elicit amyloidogenesis, we stained both MPNST cells and HSCs with Congo red (CR), a widely applied amyloid-binding fluorescent dye.^25^ Compared to HSCs, MPNST cells displayed more intense CR staining (Figure 1E), suggesting increased amyloids in malignant Schwann cells. Importantly, *HSF1* depletion further intensified CR staining in MPNST cells, but not in HSCs (Figures 1E, 1F, S1D, and S1E). Similar results were obtained by Thioflavin T staining (Figure S1F), another popular amyloid dye.^25^ Importantly, the induction of amyloids was further confirmed by ELISA using two conformation-specific antibodies (A11 and OC), which recognize soluble amyloid oligomers (AOs) and insoluble amyloid fibrils (AFs), respectively.^26,27^ Upon *HSF1* depletion, all three MPNST cell lines displayed elevated levels of A11^+^ AOs and OC^+^ AFs (Figures 1G, 1H, S1G-S1J). Again, no evident increase in amyloids was detected in HSCs (Figures 1I and 1J). Together, these findings indicate that HSF1 is required to safeguard the proteome of malignant Schwann cells and particularly to repress amyloidogenesis; on the contrary, it is dispensable for the proteome of non-transformed Schwann cells.

### Unchecked proteomic instability is tumor suppressive

It has become evident that HSF1 acts as a generic oncogenic enabler; nevertheless, a universal underlying mechanism remains undefined.

**Figure 2:**
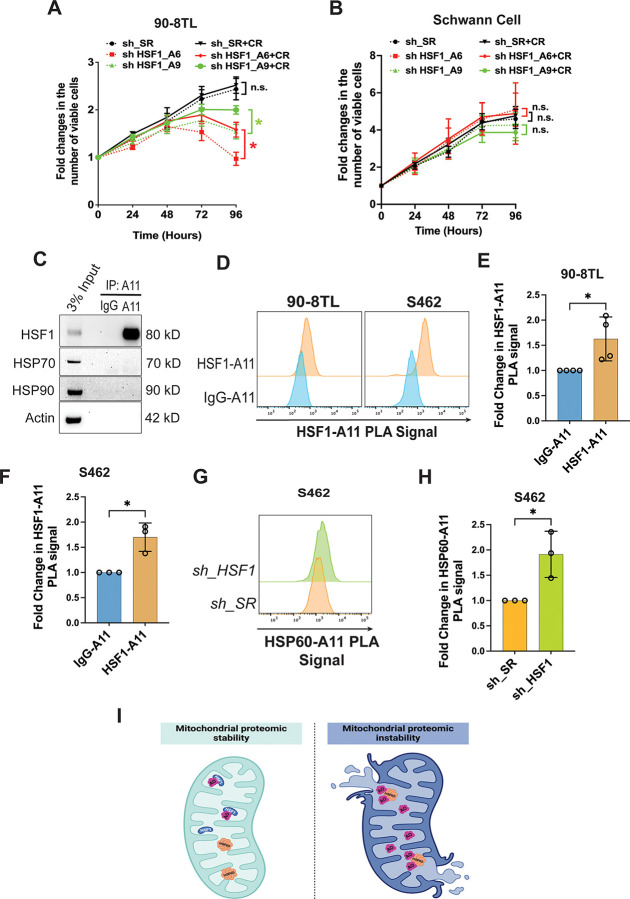
HSF1 counters the tumor-suppressive effects of amyloids in MPNST cells. (A) and (B) The growth curves of 90-8TL and primary human Schwann cells with and without *HSF1* depletion and 20 μM CR treatment (mean ± SD, n=3 independent experiments, Two-way ANOVA). (C) Co-IP of amyloid oligomers (AOs) with HSF1 in 90-8TL cells. (D)-(F) Detection of AO-HSF1 interactions by PLA flow cytometry in 90-8TL and S462 cells. Quantitation of the PLA signals using the median fluorescence intensity (mean ± SD, n=3 or 4 independent experiments, Student’s t test). (G) and (H) Detection of AO-HSP60 interactions by PLA flow cytometry in S462 cells following *HSF1* depletion (G). Quantitation of the PLA signals using the median fluorescence intensity (H) (mean ± SD, n=3 independent experiments, Student’s t test). (I) Schematic depiction of AO neuralization by HSF1 and HSP60 attack by AO following *HSF1* depletion in mitochondria.

A healthy proteome enables the translation of genotypes into phenotypes. Thus, we reasoned that preservation of proteomic stability is most likely a generic pro-oncogenic mechanism of HSF1. To test this notion, we first determined whether proteomic instability, particularly amyloidogenesis, is causally related to the cytotoxicity induced by *HSF1* depletion in cancer cells. To block amyloidogenesis we employed CR, which is known to exert anti-amyloid effects through its amyloid-binding activity.^5,28,29^ Consistent with “HSF1 addiction of cancer”, *HSF1* depletion markedly impaired the viability of MPNST cells; importantly, CR treatment partially rescued this impaired viability (Figures 2A, S2A, and S2B). In contrast, both *HSF1* depletion and CR treatment did not affect HSCs (Figure 2B), congruent with a lack of proteomic instability and amyloidogenesis in these non-transformed cells. Together, these findings support that unchecked proteomic instability, particularly amyloidogenesis, is cytotoxic and tumor suppressive. Moreover, HSF1 enables oncogenesis in part by constraining proteomic instability.

### HSF1 neutralizes soluble amyloid oligomers to protect MPNST cells

Then, how does HSF1 counter amyloids to enable the growth and survival of MPNST cells? Recently, by researching tissue overgrowth models we discovered that HSF1, surprisingly, physically neutralizes soluble AOs, thereby preventing their assault on HSP60.^30^ As an essential mitochondrial chaperone, HSP60 functions as a guardian of the mitochondrial proteome.^30–32^

We wondered whether HSF1 acts the same way in both cancer and tissue overgrowth. Co-IP experiments indicated that A11^+^ AOs physically interacted with HSF1, but not major HSPs, in MPNST cells (Figure 2C), consistent with our findings in tissue overgrowth models.^30^ This AO-HSF1 interaction was further validated by another independent technique, *in situ* Proximity Ligation Assay (PLA) (Figures 2D–2F). Importantly, *HSF1* depletion promoted the AO-HSP60 interaction (Figure 2G and 2H). Loss of *HSP60* destabilized the mitochondrial proteome.^30,32^ Thus, this AO-HSP60 interaction signifies the instigation of mitochondrial damage and cytotoxicity. Taken together, our findings reveal that HSF1 protects MPNST cells in part by neutralizing soluble AOs to safeguard HSP60 and the mitochondrial proteome (Figure 2I).

### MPNST cells react to HSF1 deficiency by activating JNK to attenuate translation

Given their dependency on HSF1, cancer cells are expected to mount certain responses to survive and adapt to the lethal HSF1 inhibition. Previously, we uncovered that in *Hsf1*-deficient mice, the stress-responsive JNK kinase becomes constitutively activated, which, in turn, disassembles mTORC1.^33^ JNK does so by phosphorylating both RAPTOR and mTOR, two core components of mTORC1. This mTORC1 inhibition diminishes protein translation, leading to reduced cell size and whole-body lean mass in *Hsf1*-deficient mice.^33^

**Figure 3:**
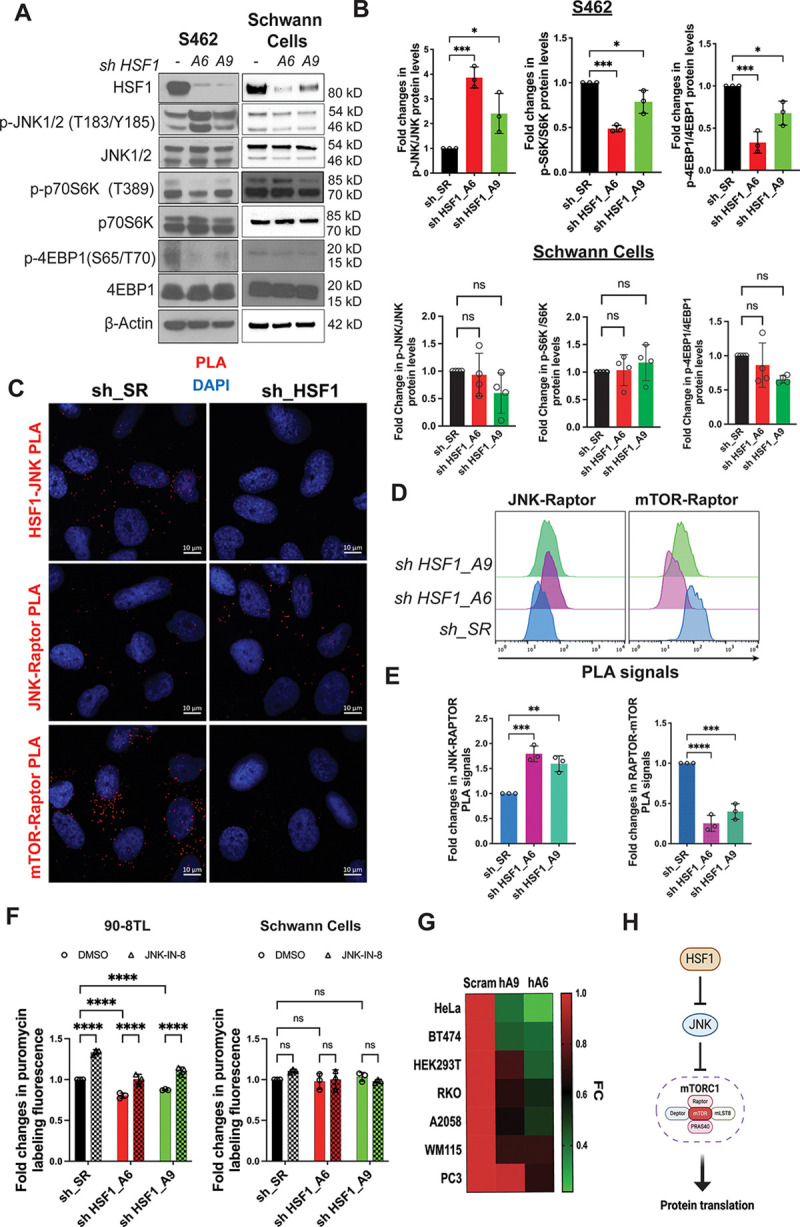
JNK senses *HSF1* deficiency to repress mTORC1 and protein translation in MPNST cells. (A) Detection of JNK activation and mTORC1 inhibition following *HSF1* depletion in S462 and primary human Schwann cells by immunoblotting. (B) Quantitation of (A) using Fiji imaging software (mean ± SD, n=3 independent experiments, One-way ANOVA). (C) Detection of HSF1-JNK, JNK-RAPTOR, and mTOR-RAPTOR interactions by PLA in S462 cells with and without *HSF1* depletion. (D) and (E) Detection and quantitation of JNK-RAPTOR and mTOR-RAPTOR interactions by PLA flow cytometry in 90-8TL cells (mean ± SD, n=3 independent experiments, One-way ANOVA). (F) Measurement of protein translation rates in 90-8TL and primary human Schwann cells with and without *HSF1* depletion by puromycin labeling, analyzed by flow cytometry. Cells were treated with 1 μM JNK-IN-8 for 24 hrs before labeling with 100 nM 6-FAM-dc-puromycin for 30 min (mean ± SD, n=3 independent experiments, Two-way ANOVA). (G) Heatmap of protein translation rates measured by puromycin labeling in diverse human cancer cell lines with and without *HSF1* depletion. (H) Schematic depiction of regulation of protein translation by HSF1.

We reasoned that cancer cells might exploit this molecular regulation for their survival and adaptation. In MPNST cells *HSF1* knockdown activated JNK, indicated by elevated JNK T183/Y185 phosphorylation; in parallel to JNK activation, there was mTORC1 inhibition, evidenced by diminished p70S6K T389 phosphorylation and 4EBP1 S65/T70 phosphorylation (Figures 3A, 3B, and S3A-S3D).^34,35^ In contrast, *HSF*1 knockdown minimally affected JNK and mTORC1 in HSCs (Figures 3A and 3B). Detected by PLA, in MPNST cells HSF1 interacted with JNK; however, *HSF1* depletion promoted JNK-RAPTOR interactions but diminished mTOR-RAPTOR interactions simultaneously (Figures 3C–3E). The mTOR-RAPTOR interaction is essential to the assembly and activity of mTORC1.^36^ Thus, these findings support that JNK senses HSF1 deficiency to repress mTORC1. Of interest, compared to non-transformed cells, this sensing mechanism appears more prominent in malignant cells.

As expected for mTORC1 inhibition, *HSF1* depletion reduced global protein translation rate in MPNST cells (Figures 3F, S3E, and S3F), detected by puromycin labeling as described previously.^33,37^ Beyond MPNST cells, this regulation of translation by HSF1 was conserved in diverse human cancer cell lines (Figure 3G). Importantly, treatment with JNK-IN-8, an irreversible JNK inhibitor,^38^ largely rescued this impaired protein translation owing to *HSF1* depletion (Figures 3F, S3E, and S3F). Interestingly, JNK inhibition augmented the basal translation rate as well. Consistent with these findings, in human cancers higher *HSF1* expression is associated with lower JNK activation (Figure S3G); in turn, lower JNK activation is accompanied by higher mTORC1 activity, reflected by higher 4EBP1 Ser65 phosphorylation (Figure S3H). In aggregate, our findings demonstrate that JNK activation is causally linked to inhibition of mTORC1 and translation. Therefore, through JNK suppression HSF1 permits robust protein translation, which is fundamental to malignant growth (Figure 3H).

### Lessening protein quantity is an adaptive response to alleviate proteomic instability

We next asked why cells respond to HSF1 deficiency by attenuating translation. While HSF1 controls protein quality via HSPs, mTORC1 governs protein quantity via translation. It would be beneficial to lower protein quantity when protein quality is jeopardized. Thus, we reasoned that this regulation might represent a cellular adaptive response to imbalanced protein quantity and quality engendered by HSF1 deficiency.

**Figure 4:**
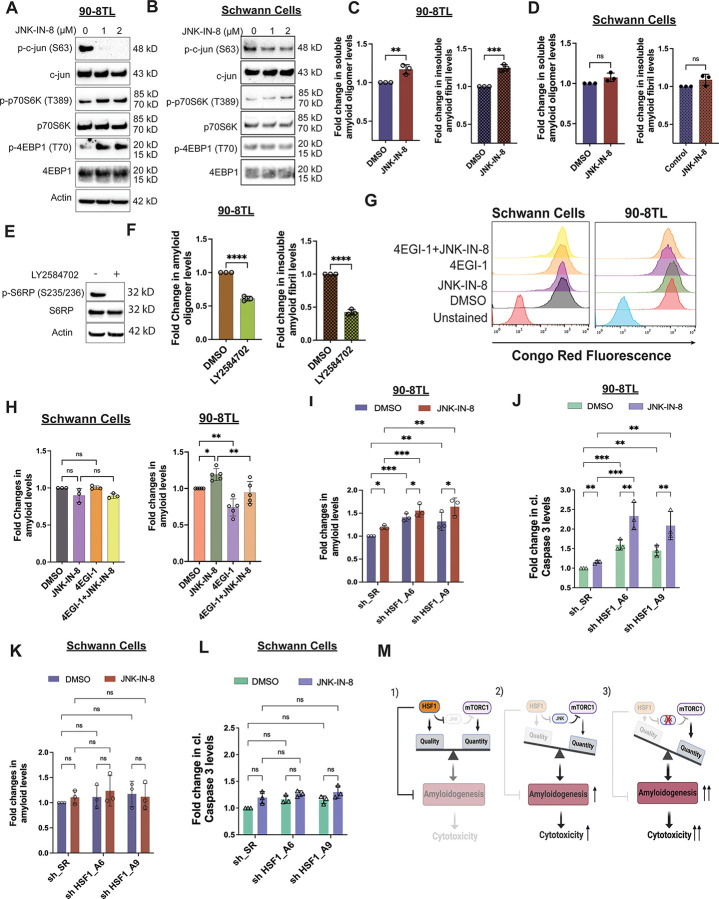
Protein translation is causally related to amyloidogenesis in MPNST cells. (A) and (B) Detection of mTORC1 stimulation following JNK inhibition in 90-8TL and immortalized human Schwann cells by immunoblotting. Cells were treated with JNK-IN-8 for 2 days. (C) and (D) Quantitation of soluble AOs and insoluble AFs by ELISA in 90-8TL and human Schwann cells with and without 3 μM JNK-IN-8 treatment for 3 days (mean ± SD, n=3 independent experiments, Student’s t test). (E) Detection of LY2584702-mediated S6K inhibition in 90-8TL cells by immunoblotting. Cells were treated with 20 μM LY2584702 for 2 days. (F) Quantitation of soluble AOs and insoluble AFs by ELISA in 90-8TL cells with and without 20 μM LY2584702 treatment for 2 days (mean ± SD, n=3 independent experiments, Student’s t test). (G) CR staining of 90-8TL and immortalized human Schwann cells with and without JNK-IN-8 and 4EGI-1 treatment, detected by flow cytometry. (H) Quantitation of (G) using the median fluorescence intensity (mean ± SD, n=3 independent experiments, One-way ANOVA). (I) Quantitation of amyloids by CR staining in 90-8TL cells with and without *HSF1* depletion and 3 μM JNK-IN-8 treatment (mean ± SD, n=3 independent experiments, Two-way ANOVA). (J) Quantitation of caspase 3 cleavage by ELISA in 90-8TL cells with and without *HSF1* depletion and 3 μM JNK-IN-8 treatment (mean ± SD, n=3 independent experiments, Two-way ANOVA). (K) Quantitation of amyloids by CR staining in immortalized human Schwann cells with and without *HSF1* depletion and 3 μM JNK-IN-8 treatment (mean ± SD, n=3 independent experiments, Two-way ANOVA). (L) Quantitation of caspase 3 cleavage by ELISA in immortalized human Schwann cells with and without *HSF1* depletion and 3 μM JNK-IN-8 treatment (mean ± SD, n=3 independent experiments, Two-way ANOVA). (M) Schematic depiction of the adaptation of MPNST cells to *HSF1* depletion by activating JNK to suppress mTORC1 and protein translation, thereby alleviating amyloidogenesis and cytotoxicity.

Proteomic imbalance disrupts proteome homeostasis and contributes to proteomic instability, which may ultimately trigger amyloidogenesis. To inflict proteomic imbalance, we first stimulated protein translation in MPNST cells by inhibiting JNK. As expected, JNK-IN-8 reduced the phosphorylation of c-JUN, a canonical substrate of JNK,^34^ in MPNST cells (Figure 4A). In support of enhanced protein translation, JNK-IN-8 further activated mTORC1 in these cells (Figures 4A and S4A); in contrast, marginal effects were observed in HSCs (Figure 4B). Congruently, JNK-IN-8 did not significantly reduce c-JUN phosphorylation in HSCs, supporting a lack of JNK activation in these non-transformed Schwann cells. Intriguingly, the heightened protein translation following JNK inhibition was accompanied by elevated amyloid levels in MPNST cells (Figures 4C and S4B). On the contrary, there were no significant changes in amyloid levels in HSCs (Figure 4D).

To determine the role of protein translation in amyloidogenesis, we chose to block translation using two pharmacological inhibitors, 4EGI-1 and LY2584702, with distinct mechanisms of action. While 4EGI-1 disrupts eIF4E-eIF4G interactions,^39^ LY2584702 inhibits p70S6K.^40^ Interestingly, both inhibitors diminished the basal amyloid levels in MPNST cells (Figures 4E,4F, and S4C), revealing a causal relationship between protein translation and amyloidogenesis. Importantly, translation inhibition also blocked the induction of amyloids by JNK-IN-8 in MPNST cells (Figures 4G, 4H, S4D, and S4E), indicating that JNK suppresses amyloidogenesis via translation inhibition. No significant effects were observed in HSCs (Figures 4G and 4H).

Considering the role of translation in amyloidogenesis, we reasoned that preventing translation attenuation would aggravate proteomic imbalance and enhance cytotoxicity in malignant cells. As expected, JNK-IN-8 further elevated the amyloid levels in MPNST cells following *HSF1* depletion, which was accompanied by exacerbated apoptosis, detected by caspase 3 cleavage (Figures 4I, 4J, and S4F).^41^ Importantly, CR treatment blocked apoptosis, detected by PARP cleavage (Figure S4G),^42^ causally implicating amyloidogenesis. In contrast, *HSF1* depletion and JNK inhibition exerted no significant impacts on non-transformed Schwann cells (Figures 4K and 4L). Thus, our findings support that proteomic imbalance in malignant cells contributes to proteomic instability and amyloidogenesis. In stark contrast, there is no or little amyloidogenesis in non-transformed cells, owing to their balanced protein quantity and quality. Moreover, through translation attenuation, malignant cells attempt to survive and adapt to proteomic imbalance (Figure 4M).

### mTORC1 stimulation aggravates proteomic imbalance and cytotoxicity induced by HSF1 inhibition

**Figure 5:**
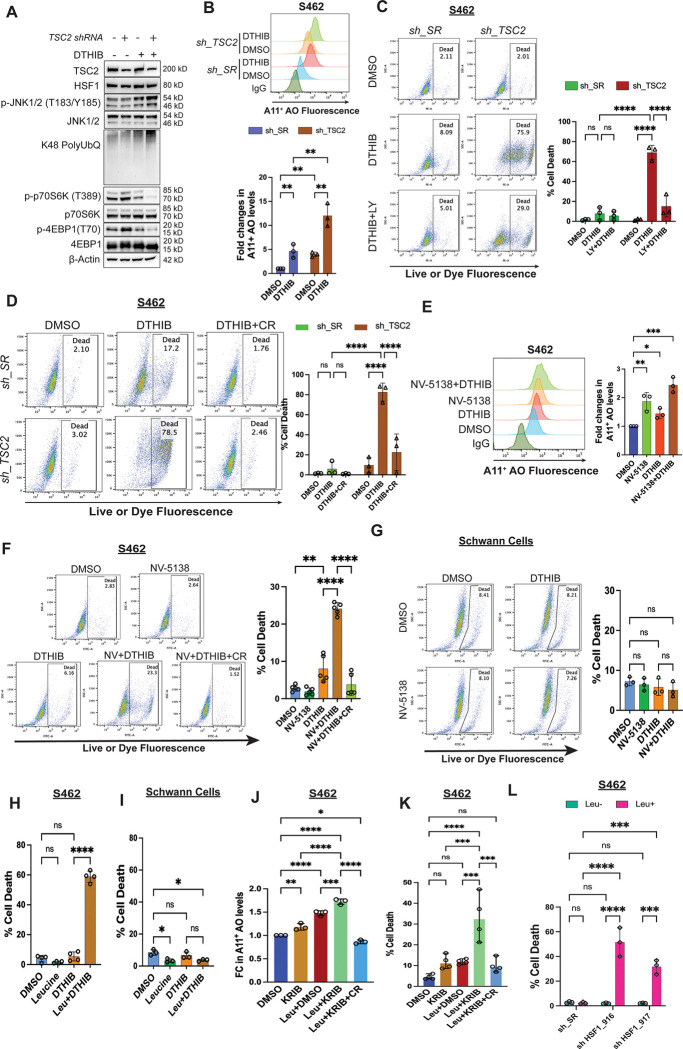
mTORC1 stimulation aggravates the amyloidogenesis and cytotoxicity elicited by HSF1 inhibition. (A) Immunoblotting detection of JNK activation, mTORC1 activity, and protein polyubiquitination in S462 cells following stable *TSC2* knockdown and/or HSF1 inhibition by 10 μM DTHIB for 3 days. (B) Quantitation of soluble AOs by flow cytometry using A11 antibody staining in S462 cells with and without *TSC2* knockdown and 10 μM DTHIB treatment (mean ± SD, n=3 independent experiments, Two-way ANOVA). (C) Quantitation of cytotoxicity by flow cytometry using Live-or-Dye stains in S462 cells with and without stable *TSC2* knockdown and DTHIB (10 μM) or combined DTHIB and LY2584702 (20 μM) treatment (mean ± SD, n=3 independent experiments, Two-way ANOVA). Cells were pretreated with LY2584702 for 1 day followed by DTHIB treatment for another 3 days. (D) Quantitation of cytotoxicity by flow cytometry using Live-or-Dye stains in S462 cells with and without stable *TSC2* knockdown and DTHIB (10 μM) or combined DTHIB and CR (30 μM) treatment (mean ± SD, n=3 independent experiments, Two-way ANOVA). Cells were pretreated with CR for 1 day followed by DTHIB treatment for another 3 days. (E) Quantitation of soluble AOs by flow cytometry using A11 antibody staining in S462 cells with and without 500 μM NV-5138 stimulation and 10 μM DTHIB treatment (mean ± SD, n=3 independent experiments, One-way ANOVA). (F) Quantitation of cytotoxicity by flow cytometry using Live-or-Dye stains in S462 cells with and without 500 μM NV-5138 stimulation and 10 μM DTHIB or combined DTHIB and CR treatment (mean ± SD, n=5 independent experiments, One-way ANOVA). (G) Quantitation of cytotoxicity by flow cytometry using Live-or-Dye stains in immortalized human Schwann cells with and without 500 μM NV-5138 stimulation and 10 μM DTHIB treatment (mean ± SD, n=3 independent experiments, One-way ANOVA). (H) Quantitation of cytotoxicity by flow cytometry using Live-or-Dye stains in S462 cells with and without 500 μM L-leucine stimulation and 10 μM DTHIB treatment (mean ± SD, n=4 independent experiments, One-way ANOVA). (I) Quantitation of cytotoxicity by flow cytometry using Live-or-Dye stains in immortalized human Schwann cells with and without 500 μM L-leucine stimulation and 10 μM DTHIB treatment (mean ± SD, n=3 independent experiments, One-way ANOVA). (J) Quantitation of soluble AOs by ELISA in S462 cells treated with and without 800 μM L-leucine and 20 μM KRIBB11 or 30 μM CR (mean ± SD, n=3 independent experiments, One-way ANOVA). (K) Quantitation of cytotoxicity by flow cytometry using Live-or-Dye stains in S462 cells treated as above (mean ± SD, n=4 independent experiments, One-way ANOVA). (L) Quantitation of cytotoxicity by flow cytometry using Live-or-Dye stains in S462 cells stably expressing inducible shRNAs with and without L-leucine stimulation. Cells were first cultured with or without 1.2 mM L-leucine for 2 days, followed by addition of 20 ng/ml doxycycline for 7 days (mean ± SD, n=3 independent experiments, One-way ANOVA).

JNK regulates numerous targets other than mTORC1. To stimulate mTORC1 and protein translation specifically, we next genetically inhibited the tumor suppressor Tuberous Sclerosis Complex subunit 2 (*TSC2*), a key negative regulator of mTORC1,^43^ in MPNST cells. As expected, knocking down *TSC2* in MPNST cells promoted phosphorylation of both p70S6K and 4EBP1, which was accompanied by elevated amyloids (Figures S5A-S5F). Like genetic *HSF1* depletion, pharmacological inhibition of HSF1 by DTHIB elevated global protein polyubiquitination and amyloids in MPNST cells (Figures 5A, 5B, and S5G), eliciting proteomic instability. The newly developed small-molecule inhibitor DTHIB directly binds to the DNA-binding domain of HSF1 and potently blocks the HSF1-mediated transcription.^44^ Compared to control cells, HSF1 inhibition by DTHIB induced considerably higher levels of amyloids in *TSC2*-deficient MPNST cells (Figure 5B). Of note, *TSC2*-deficient cells displayed more profound JNK activation and mTORC1 inhibition following DTHIB treatment, reflecting an intensified cellular response to severe proteotoxic stress. Consistently, compared to slight or intermediate cytotoxicity in control MPNST cells, detected by plasma membrane permeabilization, *TSC2*-deficient cells were highly sensitive to HSF1 inhibition (Figures 5C, 5D, and S5H-S5K). Importantly, either translation inhibition or CR treatment largely blocked this cytotoxicity (Figures 5C and 5D), causally implicating proteomic instability and amyloidogenesis. In support of a role of HSF1 in enabling *TSC2* deficiency, among patients whose tumors harbor *TSC2* deletion higher *HSF1* gene expression is correlated with shortened survival (Figure S5L).

To exclude possible mTORC1-independent effects of *TSC2* deficiency, we alternatively fed cells with NV-5138, a leucine analog currently under clinical trials as an antidepressant.^45,46^Amino acids, particularly leucine, are potent activators of mTORC1.^47^ Of interest, whereas NV-5138 is capable of activating mTORC1, it cannot be utilized for protein synthesis.^45,46^ To maximize mTORC1 stimulation *ex vivo*, cells were cultured in leucine-free medium. Like *TSC2* knockdown, NV-5138 stimulated mTORC1 and heightened amyloid levels in MPNST cells (Figures S5M and 5E); however, it only elicited pronounced cytotoxicity with concurrent HSF1 inhibition (Figure 5F). Again, CR treatment largely blocked this cytotoxicity (Figure 5F). In sharp contrast, combined NV-5138 and DTHIB treatment did not significantly impact non-transformed Schwann cells (Figure 5G). Of interest, their mTORC1 signaling was less responsive to NV-5138 stimulation (Figures S5N). We next reasoned that by directly contributing to protein quantity, L-leucine might impact the cancer proteome more profoundly. In both S462 and 90-8TL cells, L-leucine synergized with DTHIB to induce severe cytotoxicity (Figures 5H and S5O). On the contrary, no cytotoxicity was induced in non-transformed Schwann cells (Figure 5I).

To further corroborate the HSF1-dependent effects of DTHIB, we evaluated another direct small-molecule HSF1 inhibitor KRIBB11^48^, which blocks the recruitment of pTEFb likely through binding to the transactivation domain of HSF1. KRIBB11, like DTHIB, synergized with L-leucine to induce amyloids and trigger severe cytotoxicity in MPNST cells; importantly, CR treatment both diminished amyloids and blocked cell death (Figures 5J and 5K).

Furthermore, in S462 cells shRNA-mediated *HSF1* knockdown and L-leucine stimulation synergistically provoked prominent cytotoxicity as well (Figure 5L), providing a genetic validation. Thus, these findings collectively indicate that mTORC1 stimulation synergizes with HSF1 inhibition to rapidly trigger severe cytotoxicity in cancerous cells, in part by prompting unconstrained proteomic imbalance and amyloidogenesis.

### Unconstrained proteomic imbalance incites non-apoptotic, non-autophagic death

**Figure 6:**
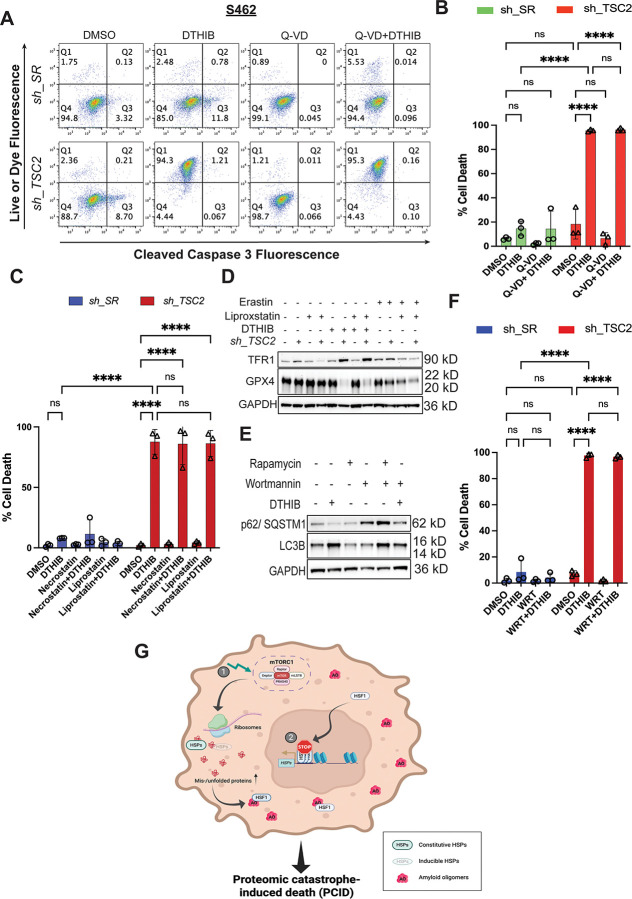
Concurrent HSF1 inhibition and mTORC1 stimulation instigate widespread non-apoptotic cell death. (A) and (B) Quantitation of cytotoxicity by flow cytometry using Live-or-Dye stains combined with cleaved caspase 3 (Asp175) antibody staining. S462 cells with and without stable *TSC2* knockdown were treated with DMSO, 10 μM DTHIB, or combined DTHIB and 30 μM Q-VD-OPh (mean ± SD, n=3 independent experiments, Two-way ANOVA). (C) Quantitation of cytotoxicity by flow cytometry using Live-or-Dye stains in S462 cells with and without stable *TSC2* knockdown. Cells were treated with 10 μM DTHIB alone or co-treated with 10 μM DTHIB and 30 μM Necrostatin-1 or 20 μM Liproxstatin-1 for 3 days (mean ± SD, n=3 independent experiments, Two-way ANOVA). (D) Immunoblotting detection of ferroptosis markers in S462 cells with and without stable *TSC2* knockdown treated with 10 μM DTHIB alone or co-treated with 10 μM DTHIB and 20 μM liproxstatin-1 for 3 days. Erastin was included as a positive control to induce canonical ferroptosis. (E) Immunoblotting detection of autophagy markers in S462 cells with stable *TSC2* knockdown treated with 10 μM DTHIB alone or co-treated with 10 μM DTHIB and 3 μM wortmannin for 3 days. Rapamycin was included as a positive control to induce autophagy. (F) Quantitation of cytotoxicity by flow cytometry using Live-or-Dye stains in S462 cells with and without stable *TSC2* knockdown. Cells were treated with 10 μM DTHIB alone or co-treated with 10 μM DTHIB and 3 μM wortmannin for 3 days (mean ± SD, n=3 independent experiments, Two-way ANOVA). (G) Schematic depiction of instigation of cell death by severe proteomic imbalance, owing to simultaneous mTORC1 stimulation (❶) and HSF1 inhibition (❷). In cancer cells, constitutive HSF1 activation provides extra chaperoning capacity to cope with elevated protein misfolding, partly due to enhanced protein synthesis and widespread genetic mutations. Nevertheless, amyloids still emerge, although at low levels. Importantly, HSF1 can neutralize highly toxic amyloid oligomers, averting lethal consequences. By contrast, HSF1 inhibition diminishes chaperoning capacity, insufficient to counterbalance the robust protein translation. mTORC1 stimulation further aggravates this proteomic imbalance, which, in turn, promotes amyloidogenesis and proteomic catastrophe. In consequence, the amounts of amyloid oligomers surpass the neutralizing capacity of HSF1, leading to cell death; nonetheless, it remains unclear how this proteomic catastrophe-induced death (PCID) occurs.

HSF1 inhibition alone induced both apoptotic and non-apoptotic death in MPNST cells, distinguished by caspase 3 cleavage; intriguingly, concurrent mTORC1 stimulation caused a striking shift towards non-apoptotic cell death, characterized by cell membrane permeabilization without caspase 3 cleavage (Figures 6A and 6B).

To date, several modes of non-apoptotic cell death have been described, including pyroptosis, necroptosis, ferroptosis, and autophagic death, all of which could result in loss of cell membrane integrity. Corroborating a non-apoptotic mechanism, the pan-caspase inhibitor Q-VD-OPh did not prevent the membrane permeabilization in *TSC2*-deficient MPNST cells treated with DTHIB (Figures 6A and 6B).^49^ However, it did block the apoptosis in control MPNST cells treated with DTHIB (Figures 6A and S6A). As Q-VD-OPh inhibits caspase 1 and no caspase 1 cleavage was detected (Figure S6B),^50^ a key driver of pyroptosis,^51^ this result does not support a pyroptotic cell death either. Similarly, HSF1 inhibition did not induce necroptosis in *TSC2*-deficient MPNST cells, evidenced by the lack of MLKL Ser358 phosphorylation (Figure S6B), an essential event executing necroptosis.^52^ Congruently, the necroptosis inhibitor necrostatin-1 exerted no effects on the cell membrane disruption (Figures 6C and S6C).^52^

Intriguingly, HSF1 inhibition diminished glutathione peroxidase 4 (GPX4) but increased transferrin receptor 1 (TFR1), which was accompanied with markedly elevated lipid peroxidation, in *TSC2*-deficient MPNST cells (Figures 6D and S6D). All these changes are indicative of ferroptosis.^53,54^ Although in *TSC2*-deficient cells liproxstatin-1, a potent ferroptosis inhibitor,^53,54^ reversed the elevation of TFR1 elicited by the ferroptosis inducer erastin, it neither blocked the marked increase in TFR1 nor stopped the membrane disruption induced by DTHIB (Figures 6C, 6D, and S6C). This is not due to the defectiveness of necrostatin-1 and liproxstatin-1, as both inhibitors potently blocked necroptosis and ferroptosis induced in HT-29 cells, respectively (Figure S6E). HSF1 inhibition also elicited autophagy, evidenced by diminished p62/SQSTM1 and elevated LC3B-II (Figure 6E).^55^ To determine whether autophagic cell death was implicated, we employed the class III PI3K inhibitor wortmannin to block autophagy.^56^ As expected, wortmannin effectively prevented the decreases in p62/SQSTM1 and LC3B-II induced by rapamycin in *TSC2*-deficient MPNST cells (Figure 6E), indicating successful blockade of autophagy. Nevertheless, wortmannin failed to block the cell membrane disruption in *TSC2-*deficient cells treated with HSF1 inhibitors (Figures 6F and S6F). These findings suggest that both ferroptosis and autophagy are the consequences, rather than the causes, of cell membrane disruption. Collectively, our findings support that uncontained proteomic instability and ensuing amyloidogenesis potently trigger cell death, which is neither apoptotic nor autophagic (Figure 6G). Moreover, several common modes of programmed necrosis are not deeply implicated as well. The precise nature of this proteomic catastrophe-induced death (PCID), nonetheless, remains to be fully delineated.

### Unconstrained proteomic imbalance suppresses *in vivo* tumor growth

**Figure 7:**
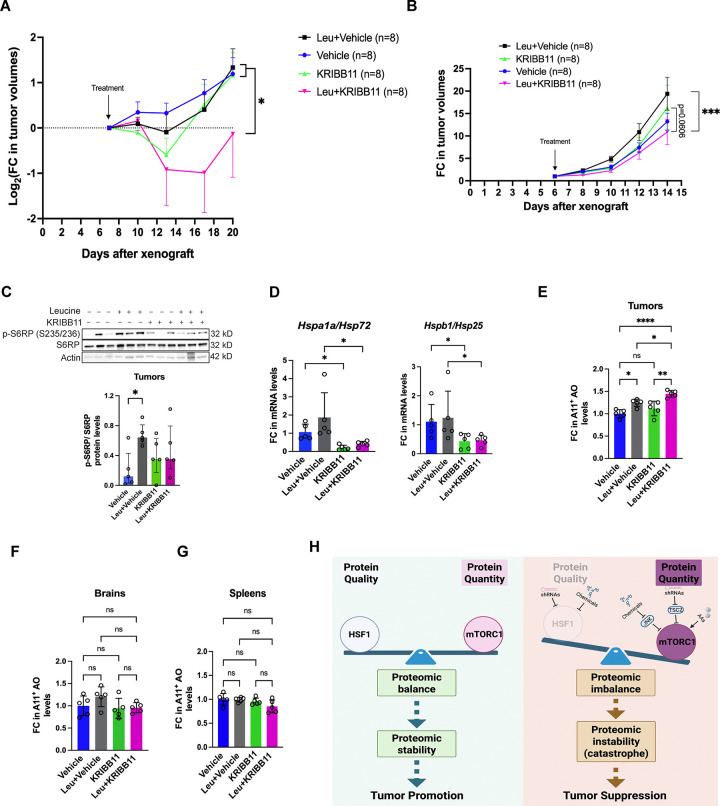
Concurrent HSF1 inhibition and mTORC1 stimulation suppress *in vivo* tumor growth. (A) Combined KRIBB11 treatment and L-leucine stimulation impaired the *in vivo* growth of xenografted S462 human MPNST cells in NIH-III nude mice (mean ± SEM, n=8 mice per group, log_2_ transformation for Two-way ANOVA). (B) Combined KRIBB11 treatment and L-leucine stimulation impeded the *in vivo* growth of xenografted B16-F10 murine melanoma cells in C57BL/6J mice (mean ± SEM, n=8 mice per group, log_2_ transformation for Two-way ANOVA). (C) Quantitation of phospho-S6RP by immunoblotting in treated melanomas. Images show representative 3 tumors each group (mean ± SEM, n=5 tumors per group, One-way ANOVA). (D) Quantitation of *Hsp72* and *Hsp25* mRNAs in treated melanomas by RT-qPCR (mean ± SD, n=5 tumors per group, One-way ANOVA). (E) Quantitation of soluble AOs in treated B16F10 melanomas by ELISA ((mean ± SD, n=5 tumors per group, One-way ANOVA). (F) and (G) Quantitation of soluble AOs in brains and spleens from treated C57BL/6J mice by ELISA (mean ± SD, n=5 brains or spleens per group, One-way ANOVA). (H) Schematic depiction of the concept of provoking proteomic catastrophe to combat malignancy. On the one hand, in cancer cells, mTORC1 is inevitably activated to stimulate protein translation, markedly augmenting protein quantity. On the other hand, the extra chaperoning capacity governed by HSF1, albeit dispensable for normal life, becomes necessary to ensure sufficient protein quality in cancer cells, thereby counterbalancing augmented protein quantity and suppressing proteomic instability. Thus, proteomic balance promotes malignant growth. By contrast, disrupting proteomic balance, through HSF1 inhibition, is sufficient to elicit proteomic instability and tumor suppression. However, simultaneous mTORC1 stimulation can remarkably drive proteomic imbalance, causing proteomic catastrophe that leads to tumor suppression.

Due to the limited *in vivo* bioavailability of DTHIB in our mouse models, we focused on KRIBB11 for *in vivo* studies. Consistent with *ex vivo* findings, combined L-leucine stimulation and KRIBB11 administration significantly impaired the *in vivo* growth of xenografted human S462 MPNST cells in immunocompromised mice (Figure 7A). Despite this overall impact, 2 out of 8 tumors did not respond to the combination treatment (Figure S7A), creating large variations and right-skewed distribution. Of note, without leucine stimulation KRIBB11 only marginally affected tumor growth; by contrast, in the presence of leucine KRIBB11 did evidently slow tumor exponential growth rates and delay tumor volume doubling time (Figures S7B). In addition, mice receiving this combination treatment tended to survive better as well (Figure S7C).

Next, we asked if this therapeutic strategy could be applied broadly. To this end, we established a syngeneic mouse xenograft model of melanoma, where the murine melanoma B16-F10 cells were transplanted into C57BL/6J mice. Like in the MPNST model, KRIBB11 combined with L-leucine stimulation also impeded the tumor growth in this fast-growing tumor model (Figures 7B and S7D). As expected, L-leucine stimulated mTORC1 signaling, evidenced by increased S6RP phosphorylation (Figure 7C). KRIBB11 reduced the transcription of *Hspa1a/Hsp72* and *Hspb1/Hsp25* in melanomas (Figure 7D), confirming a successful inhibition of HSF1. Importantly, combined KRIBB11 administration and leucine stimulation markedly induced amyloids in tumors (Figure 7E). By stark contrast, no amyloids were induced in normal tissues including brains and spleens (Figures 7F and 7G). Treatments did not cause loss of body weights in mice (Figure S7E). In aggregate, these *in vivo* findings from two distinct mouse models support that unconstrained proteomic imbalance, provoked by combined HSF1 inhibition and mTORC1 stimulation, causes proteomic catastrophe that is tumor suppressive (Figure 7H).

## DISCUSSIONS

Genomic instability has been broadly recognized as a driver of oncogenesis. Contrastingly, the implications of proteomic instability in cancer remain obscure.

### Proteomic instability is tumor suppressive but inevitable

Proteomic instability does occur in cancer, evidenced by elevated protein polyubiquitination, aggregation, and even amyloidogenesis. In cancer, proteomic instability can be ascribed to several mechanisms, including protein oxidation by reactive oxygen species, protein misfolding owing to genetic mutations, and augmentation of protein dosage caused by aneuploidy.^1,3^ Revealed by our recent studies, uncontrolled protein translation also contributes to amyloidogenesis. This is not entirely surprising, as it has been estimated that up to 30% of all newly synthesized polypeptides are misfolded and subjected to proteasomal degradation.^57^ Imaginably, aberrant protein synthesis would increase the chance of producing protein aggregates and even amyloids, especially considering numerous genetic mutations affecting protein folding in cancer cells. Dysregulated protein translation, ironically, is fundamental to malignant growth, making proteomic instability and amyloidogenesis inevitable.

At the cellular level, amyloids are toxic to both neurons and cancer cells. At the organismal level, amyloidogenesis is neurodegenerative but tumor suppressive. Mechanistically, our studies demonstrate that soluble AOs can directly attack the guardian of the mitochondrial proteome HSP60. Of course, mechanisms other than targeting HSP60 exist as well. Moreover, it remains to be elucidated what other proteins comprise cancer-associated amyloids, in addition to the Alzheimer’s disease-associated Aβ.

### HSF1 contains proteomic instability to enable malignancy

The multifaceted pro-oncogenic roles of HSF1 have been ascribed to a wide array of underlying mechanisms; nonetheless, a universal one remains to be defined.

As an inevitable price of malignancy, proteomic instability must be contained by cancer cells to avert tumor suppression and sustain malignant phenotypes. Now, our findings reveal that HSF1 contains proteomic instability, particularly amyloidogenesis, constituting a generic pro-oncogenic mechanism. It is worth noting that HSF1 only contains and mitigates, but cannot eradicate, amyloidogenesis, as uncontrolled protein translation is a direct cause of amyloidogenesis in cancer cells. This dependency on HSF1 to contain proteomic instability partly underlies the phenomenon of “HSF1 addiction of cancer”.

Our studies further support that HSF1 contains amyloidogenesis via at least two mechanisms, direct and indirect. On the one hand, through induction of HSPs, HSF1 ensures appropriate protein folding to maintain a healthy proteome, thereby preventing the emergence of amyloids. This is supported by the finding that HSF1 inhibition heightens protein ubiquitination, aggregation, and amyloidogenesis. On the other hand, HSF1 can neutralize soluble AOs directly once they emerge. Under this context, HSF1 inhibition would unleash AOs to assault HSP60, inflicting mitochondrial damage and toxicity. Thus, in cancer cells HSF1 inhibition both enlarges the quantity of amyloids and, more importantly, renders them toxic.

### mTORC1 signaling becomes tumor suppressive in the absence of HSF1

Given the dependency of malignant growth on protein translation, unsurprisingly, mTORC1 signaling is hyperactive in human cancers, which is, canonically, considered oncogenic.^58,59^ Unexpectedly, our current studies reveal that mTORC1 signaling becomes tumor suppressive in the absence of HSF1, owing to profound disproportion between protein quantity and quality. This unconstrained proteomic imbalance, in turn, exacerbates proteomic instability and tumor-suppressive amyloidogenesis. The notable blockage of cytotoxicity by either translation inhibitors or CR lends strong support to this model. Conceptually, HSF1 empowers the oncogenic potential of mTORC1 signaling, reinforcing HSF1 as an oncogenic enabler.

### Provoking proteomic catastrophe exemplifies a next-generation therapeutic concept

Currently popular chemo-and radiotherapies mainly target the cancer genome, causing DNA damage. An emerging horizon in cancer therapy is targeting the cancer proteome. To date, much effort has been devoted to inhibiting individual HSP families, such as HSP70 and HSP90.^60,61^ However, every cell is equipped with both constitutive and inducible HSPs. While the former constitute the basal chaperoning capacity that is essential to life, the latter supply the extra chaperoning capacity, which is dispensable for normal life but required under stressful conditions. Their structural and functional resemblances pose the biggest challenge for small molecules to distinguish these cognate HSPs. Moreover, multiple families of HSPs, including HSP100, HSP90, HSP70, HSP60, HSP40, and small HSPs, exist inside cells. Targeting single HSP family would have limited therapeutic effects. Thus, new approaches or directions are warranted to overcome these drawbacks.

Our and others’ studies collectively support that malignant cells constantly endure intrinsic proteotoxic stress, rendering HSF1 constitutively active. In turn, cancer cells become reliant on the extra chaperoning capacity, conferred by HSF1, for their growth and survival. Thus, only eliminating this extra chaperoning capacity but sparing the basal one would be an ideal strategy to combat malignancy. Together, these concepts provide strong rationales for targeting HSF1 in cancer. It is exciting to witness the recent launch of HSF1 inhibitors into clinical trials.^62^

In reaction to HSF1 inhibition, unsurprisingly, cancer cells attenuate their protein synthesis. This adaptive response underscores the importance of balanced protein quantity and quality to cellular fitness, a key concept inspiring us to explore driving proteomic imbalance for cancer therapy. Of note, breaking this adaptation may impede tumor evolution.

The widespread hyperactivation of mTORC1 in human cancers makes it an attractive therapeutic target. Hence, an array of mTOR inhibitors have been developed, several of which are actively under clinical evaluation.^59,63^ Thus far, these inhibitors have displayed mostly cytostatic effects and less than expected therapeutic efficacies.^63,64^ Considering the essentiality of protein translation to life, profound mTORC1 inhibition would inflict undesirable toxicities in normal cells and tissues. In fact, mTORC1 inhibitors have long been known as immunosuppressants.^63,65^

Opposite to the prevailing tactic in cancer therapy, we stimulated mTORC1 to drive proteomic imbalance. Counterintuitive mTORC1 stimulation, when combined with HSF1 inhibition, engendered catastrophic proteomic instability in cancer and elicited tumor suppression (Figure 7G). This PCID offers a wide therapeutic window, largely due to the lack of proteomic instability and amyloidogenesis in primary cells and tissues. Conceivably, mTORC1 stimulation boosts production of wild-type proteins in primary cells, which can be easily managed without impacting well-preserved proteomic stability; in sharp contrast, it creates an enormous problem for the already stressed, fragile cancer proteome through synthesis of copious mutant, misfolded proteins. The adaptive response, noteworthily, still arises from concurrent mTORC1 stimulation and HSF1 inhibition in cancer. It is expected that additional, specific blockade of the JNK-mediated mTORC1 repression would further exacerbate proteomic imbalance, impelling cancerous cells to extinction. It remains possible that leucine stimulation could exert further mTORC1-independent effects, contributing to tumor suppression. Transcription factors, traditionally, have been regarded as “undruggable” targets for small molecules. While both DTHIB and KRIBB11 epitomize the prototypes of direct HSF1 inhibitors, their inadequate *in vivo* bioactivities render them unsuitable for clinical applications at the current state. Nevertheless, we herein accomplished the proof-of-concept experiments to demonstrate the feasibility of propelling proteomic catastrophe in cancer. This next-generation therapeutic concept may be applicable to a broad spectrum of human cancers and, with the advent of clinic-relevant molecules, holds promise for combating malignancy.

## EXPERIMENTAL MATERIALS AND METHODS

### Cells, Chemicals, and reagents

S462 (female) and 90-8TL (female) cells were kindly provided by Dr. Karen Cichowski. sNF96.2 cells (male) and immortalized human Schwann cells (hTERT ipn02.3 2l, female) were purchased from ATCC. Primary human Schwann cells (Cat#1700, ScienCell Research Laboratories) were grown on poly-L-lysine coated T-75 flasks and maintained in complete Schwann Cell Medium (Cat#1701, ScienCell Research Laboratories). B16-F10-luc2 (male) cells were purchased from ATCC (cat#CRL-6475-LUC2). All cell lines, except 90-8TL and HSCs (no known STR profiles available), were authenticated by ATCC. All cell lines, except primary human Schwann cells, were maintained in DMEM supplemented with 10% HyClone bovine growth serum and 1% penicillin–streptomycin. Cells were maintained in an incubator with 5% CO2 at 37 °C.

The following chemicals were purchased from commercial sources: JNK-IN-8 (Cat#S4901, Selleckchem), 6-FAM-dC-puromycin (cat#NU-925-6FM, Jena Bioscience), Congo red (cat#B2431014, Thermo Scientific Chemicals), Thioflavin T (cat#211760050, Thermo Scientific Chemicals), LY2584702 (cat#S7698, Selleckchem), 4EGI-1 (cat#S7369, Selleckchem), and DTHIB (cat# HY-138280, MedChemExpress), KRIBB11 (cat#HY-100872, MedChemExpress), KRIBB11 (cat#T3652, TargetMol Chemicals), Q-VD-OPh (cat#HY-12305, MedChemExpress), necrostatin-1 (cat#HY-15760, MedChemExpress), Liproxstatin-1 (cat#HY-12726, MedChemExpress), wortmannin (cat#HY-10197, MedChemExpress), rapamycin (cat#HY-10219, MedChemExpress), erastin (cat#HY-15763, MedChemExpress), MHY1485 (cat#HY-B0795, MedChemExpress), NV-5138 HCl (cat#HY-114384B, MedChemExpress), and Liperfluo (cat#L248, Dojindo Laboratories), dimethylacetamide (cat#T19439, TargetMol Chemicals Inc.), PEG300 (cat#T7022, TargetMol Chemicals Inc.), and Captisol (cat#RC-0C7-100, CyDex Pharmaceuticals).

Rabbit phospho-4EBP1 S65/T70 antibody (cat#sc-12884-R), mouse monoclonal HSF1 (E-4, cat#sc-17757) antibody, rabbit HSF1 antibody (H-311, cat#sc-9144), mouse monoclonal JNK (D-2, cat#sc-7345), rabbit JNK1/3 (C-17, cat#sc-474) antibody were purchased from Santa Cruz Biotechnology; antibodies for phosphor-4EBP1 Thr70 (cat#9455), phospho-4EBP1 Thr37/46 (236B4, cat#2855), phospho-p70S6K T389 (108D2, cat# 9234), phosphor-S6RP Ser235/236 (D57.2.2E, cat#4858), p70 S6K (49D7, cat#2708), 4EBP1 (53H11, cat#9644), S6RP (5G10, cat#2217), mTOR (7C10, cat#2983), phospho-JNK1/2 T183/Y185 (81E11, cat#4668), JNK2 (56G8, cat#9258), c-Jun (60A8, cat#9165), phospho-c-Jun Ser63 II (cat#9261), PARP (cat#9542), Tuberin/TSC2 (D93F12, cat#4308), SQSTM1/p62 (D1Q5S, cat#39749), cleaved caspase 3 (Asp175) (5A1E, cat#9664), caspase 1(D7F10, cat#3866), gasdermin D (E9S1X, cat#39754), phosphor-MLKL Ser358 (D6H3V, cat#91689), GPX4 (E5Y8K, cat#59735), and transferrin receptor/CD71 (H68.4, cat#46222) antibody were purchased from Cell Signaling Technologies; mouse b-actin antibody-HRP (Cat#A00730) conjugates were from GenScript. Anti-K48-specific ubiquitin antibody (Apu2, cat#05-1307) and mouse monoclonal anti-RAPTOR antibody (1H6.2, cat#05-1470) were purchased from EMD Millipore. Mouse monoclonal anti-JNK1 (C-terminal region) antibody (cat#JM2671) was purchased from ECM Biosciences. Anti-amyloid oligomers (A11, cat#SPC-506D) and anti-amyloid fibrils (OC, cat#SPC-507D) antibodies were purchased from StressMarq Biosciences Inc. Mouse monoclonal anti-HSP60 antibody (LK-1, cat#ADI-SPA-806) was purchased from Enzo Life Sciences Inc. b-Actin (cat#GTX629630), GAPDH (cat#GTX627408), a-tubulin (GT114, cat#GTX628802), and LC3B antibody (cat#GTX127375) was purchased from GeneTex Inc.

## METHOD DETAILS

### Lentiviral Particle Production and Transduction of lentiviral shRNAs

Lentiviral pLKO.1 shRNA plasmids targeting human *HSF1* were described previously^10^: TRCN0000007480 (hA6) and TRCN0000007483 (hA9). The sequence of control hairpins targeting a scrambled sequence with no known homology to any human genes is 5′-CCTAAGGTTAAGTCGCCCTCG-3′. The pLKO.1 shRNA plasmid targeting human *TSC2* was a gift from Do-Hyung Kim (Cat#15478, Addgene). Lentiviral pLKO shRNA plasmids were transiently co-transfected individually along with plasmids encoding ΔVPR and VSV-G into HEK293T cells using TurboFect^™^ (Cat#R0531, Thermo Scientific). Viral supernatants were collected after centrifugation at 1,000 rpm for 15 min. Viral transduction was achieved by incubating target cells with viral supernatants containing 10μg/ml polybrene (Cat#TR-1003-G, Millipore-Sigma) for 24h. For stable knockdown of *TSC2,* lentivirus particles were prepared using pLKO shRNA plasmids targeting human *TSC2*. Scrambled shRNA was used for stable control cells. After 4 days of transduction, cells were gradually selected using 1–4 μg/ml puromycin for 2 weeks.

### Soluble and Insoluble Protein Fractionation

Equal numbers of cells were incubated with cold cell-lysis buffer (100 mM NaCl, 30 mM Tris-HCl pH 7.6, 1% Triton X-100, 20 mM sodium fluoride, 1mM EDTA, 1mM sodium orthovanadate, and 1x Halt^™^ protease inhibitor cocktail) on ice for 20 min. The crude lysates were first centrifuged at 500xg for 5 min at 4℃. The supernatants from low-speed (500xg) centrifugation were transferred to a new Eppendorf tube. The first pellets were resuspended in DNA digestion solution (2 units DNase I, 1% Triton X-100, 10 mM Tris-HCl, 2.5 mM MgCl2, 0.5 mM CaCl2, pH 7.6) for 30 min at RT. An equal volume of 1xPBS with 2% SDS was then added to resuspend the first pellets for additional 30 min at RT. Following the centrifugation at 17,000xg for 10 min at RT, the supernatants were removed. The supernatants from low-speed centrifuge were added back to the second pellets, followed by centrifugation at 17,000xg for 15 min at 4℃. The final supernatants and pellets from high-speed (17,000xg) centrifugation were collected as detergent-soluble and -insoluble fractions, respectively. Insoluble fractions were further sonicated in 1xPBS with 2% SDS at high intensity using a Bioruptor^®^ Sonication Device (Diagenode) for SDS-PAGE.

### ThT and CR Staining for Flow Cytometry

After washing with 1xPBS, cells were fixed with 4% formaldehyde at RT for 10 min. Following fixation and 1xPBS washing, cells were resuspended in 1 ml permeabilization buffer (1xPBS with 0.5% Triton X-100 and 3 mM EDTA) and incubated on ice for 30 min. Following washing with 1xPBS, Cells were stained with 10 μM ThT or CR dissolved in 1xPBS for 5 min. Cells were washed and resuspended in PBS and fluorescence signals were measured by a FACSCalibur, LSRFortessa X-20 or LSR II flow cytometer (BD Biosciences).

### Amyloid Oligomer and Fibril Quantitation by Direct ELISA and Flow Cytometry

To quantitate soluble amyloid prefibrillar oligomers, soluble cell lysates diluted in 1xPBS from equal numbers of cells were coated on each well in a 96-well ELISA plate at 4℃ overnight followed by blocking (5% non-fat milk in TBS-0.05% Tween^®^ 20) at RT for 1 hr. Each well was incubated with 100 μl amyloid oligomer antibodies (A11, 1:1,000 diluted in the blocking buffer) at RT for 2 hr. After washing with TBS-T, goat anti-rabbit secondary Ab HRP conjugates (1:1,000 diluted in blocking buffer) were added to each well and incubated at RT for 1 hr. Following washing, 100 μl 1-Step Ultra TMB-ELISA substrates (Cat#34029, Thermo Scientific) were added to each well. After adding 100μl Stop solution, absorbance was measured at 450 nm using a SpectraMax iD5 microplate reader (Molecular Devices).

To quantitate amyloid fibrils, detergent-insoluble fractions from equal numbers of cells were extracted as described in Soluble and Insoluble Protein Fractionation. The final pellets were collected as detergent-insoluble fractions and solubilized by sonication for 15 min in 1xPBS with 2% SDS. Solubilized proteins diluted in 1xPBS were added to each well and incubated at 37℃ without cover overnight to air dry the wells. The following steps were identical to the oligomer detection except for the use of amyloid fibril antibodies (OC) as the primary Ab.

For amyloid oligomer and fibril quantification by flow cytometry, cells were collected by trypsinization and fixed in 4% Formaldehyde at RT for 10 min. Following permeabilization using 0.3% Triton-X-100, samples were incubated with primary antibodies A11-FITC (1:100 in 5% BSA, StressMarq Biosciences, cat# SPC-506D-FITC) or OC-PerCP (1:100 in 5% BSA, StressMarq Biosciences, cat# SPC-507D-PCP) at RT for 3 hrs. The cells were washed with PBS and analyzed using flow cytometry.

### Measurement of Cell Viability

Cell viability was determined using the CellTiter-Blue^®^ Cell Viability Assay (cat#G8080, Promega). Cells were grown in 96 well plates (1×10^4^ per well) for 24h. A 20μl CellTiter-Blue^®^ reagent was added to 100μl of media in each well. The plates were incubated for 2h at 37°C and the fluorescence was recorded at 560/590 nm. The reagents were discarded, and the wells were washed with PBS before adding fresh media. Similarly, subsequent readings were recorded at 24h intervals till 96h time point.

### Proximity Ligation Assay

Cells were fixed with 4% formaldehyde in PBS for 15 min at RT. After blocking with 5% normal goat serum in PBS with 0.3% Triton X-100, primary antibodies 1:100 diluted in blocking buffer were incubated with fixed cells overnight at 4°C. Following washing with PLA wash Buffer A, samples were incubated with 1:5 diluted Duolink^®^ in Situ PLA Probe anti-rabbit Plus (cat#DUO92002, Sigma-Aldrich) and anti-mouse Minus (cat#DUO92004, Sigma-Aldrich) at 37°C for 1 hr. Subsequently, ligation (30 minutes, 37°C), rolling circle amplification, and detection (120 minutes, 37°C) were performed using the Duolink^®^ In Situ Detection Reagents Red (cat#DUO92008, Sigma-Aldrich). Nuclei were counterstained with Hoechst 33342. PLA signals were documented by a Zeiss LSM780 confocal microscope (Carl Zeiss). For PLA flow cytometry, an equal number of cells were collected in microcentrifuge tubes by trypsinization and fixed with 4% formaldehyde in PBS for 10 min at RT. Subsequent PLA steps were followed as described earlier. The PLA signals were quantified by a FACSCalibur flow cytometer (BD Biosciences).

Mouse anti-JNK1 (JM2671) and rabbit anti-HSF1 (H-311) antibodies were used for JNK-HSF1 PLA. Rabbit anti-JNK1/3 (C-17) and mouse anti-RAPTOR (1H6.2) antibodies were used for JNK-RAPTOR PLA. Rabbit anti-mTOR (7C10) and mouse anti-RAPTOR (1H6.2) antibodies were used for mTOR-RAPTOR PLA. Mouse anti-HSF1 (E-4) and rabbit anti-AO (A11) antibodies were used for HSF1-AO PLA. Rabbit anti-AO (A11) and mouse anti-HSP60 (LK1) antibodies were used for AO-HSP60 PLA.

### Measurement of global protein translation

Live cells were incubated with 100 nM 6-FAM-dC-puromycin in complete culture medium for 30 min and analyzed by a FACSCalibur flow cytometer (BD Biosciences).

### Immunoblotting and Immunoprecipitation

Whole-cell protein extracts were prepared in cold cell-lysis buffer (100 mM NaCl, 30 mM Tris-HCl pH 7.6, 1% Triton X-100, 20 mM sodium fluoride, 1mM EDTA, 1mM sodium orthovanadate, and 1x Halt^™^ protease inhibitor cocktail). Proteins were transferred to nitrocellulose membranes. Following incubation with the blocking buffer (5% non-fat milk in 1x TBS-T) for 1 hour at RT, membranes were incubated with primary antibodies (1:1,000 dilution in the blocking buffer) overnight at 4 °C. After washing with 1xTBS-T for 3 times, membranes were incubated with peroxidase-conjugated secondary antibodies (1: 3,000 diluted in the blocking buffer) at RT for 1 hr. Signals were detected using SuperSignal^™^ West chemiluminescent substrates. For normal Co-IP, proteins were extracted using a QSonica Q125 sonicator (total process time: 15S, pulse-on time: 5S, pulse-off time: 10S, output intensity: 30%) in 1x sonication buffer (20 mM Tris, 20 mM NaCl, 1 mM EDTA, 20 mM β-glycerol-phosphate, 20 mM sodium fluoride, 4 mM sodium orthovanadate, and 1 mM DTT pH7.4, supplemented with Halt protease inhibitor cocktail). Total 1 mg whole cell lysates were incubated with A11 antibodies at 4 °C overnight. Normal rabbit IgG were used as the negative controls. The primary antibody was precipitated using Protein G magnetic beads. After washing with the lysis buffer for 3 times, beads were boiled in 1x loading buffer for 5 min before loading on SDS-PAGE. EasyBlocker (Cat#GTX425858, GeneTex) was used for blocking and anti-HSF1 (H-311) primary antibody (cat#sc-9144, Santa Cruz Biotechnology) incubation (1:1,000, O/N at 4°C). The blot was incubated with EasyBlot anti-Rabbit IgG HRP conjugated antibody (cat#GTX221666-01, GeneTex) for 1 hour. Chemiluminescent signals were captured by an iBright^™^ FL1000 imaging system (ThermoFisher Scientific).

### Live-Or-Dye^™^ Dead Cell Staining

Cells were cultured in DMEM for 48 hours before being treated with inhibitors for the required periods and collected by trypsinization. 1×10^6^ cells/ml cells were stained with Live-or-Dye^™^ fixable dead cell dyes (1:1,000 dilution, cat#32003, Biotium) at RT for 30 min, protected from light. Stained cells were washed once with PBS and analyzed by flow cytometry. For co-staining with cleaved caspase 3 antibody, stained cells were fixed with 4% formaldehyde (10 min, RT) and permeabilized with 0.3% Triton-X-100 in PBS (10 min, RT). The cells were stained by Cleaved Caspase 3 antibody (Asp175) (5A1E) (1:1,000, cat#9664, Cell Signaling Technology) followed by goat anti-Rabbit IgG Alexa Fluor^™^ 488 conjugated (1:1,000, cat#A-11034, ThermoFisher Scientific) secondary antibodies. The samples were analyzed by flow cytometry.

### Inhibitor treatments

Treatments with DTHIB, Q-VD-OPh, Liproxstatin-1, Necrostatin-1, and Wortmannin were done for 72 hours. For Congo Red and LY-2584702 treatments, cells were pretreated for 24h followed by DTHIB for an additional 72h. For treatments with NV-5138, cells were pretreated for 48 hours in a leucine-free DMEM (cat#226-024, Crystalgen) before adding DTHIB for an additional 72 hours.

### Animal studies

All animal experiments described here were approved by the institutional Animal Care and Use Committee (ACUC) of the National Cancer Institute, Frederick. Experiments on all animals were conducted following the recommendations of the Guide for the Care and Use of Laboratory Animals (National Academies Press, 2011). Animals were housed and maintained on a 12-hour light/12-hour dark cycle at temperatures between 20°C and 27°C and humidity levels of 30% to 70%, with food and water *available ad libitum*.

To establish the MPNST xenograft model, 9×10^6^ S462 MPNST cells (female) suspended with 30 % Matrigel were subcutaneously implanted the right flanks of 8-week-old female NIH-III nude mice (Charles River Laboratories). After 7 days, xenografted mice were assigned to 4 groups, eight each group, by stratified randomization based on tumor sizes (between 72 and 199 mm^3^). Mice were treated with KRIBB11 (65mg/kg, i.p. injection daily, cat#HY-100872, Lot#317606, Medchemexpress LLC) with and without 2% L-leucine dissolved in drinking water stored in amber-colored bottles. KRIBB11 was freshly dissolved daily in the vehicle (final concentration:10% dimethylacetamide, 50% PEG300, 20% Captisol, and 20% sterile saline).

To establish the syngeneic melanoma model, 2 × 10^6^ B16-F10-luc2 cells (male) mixed with Matrigel (1:1 ratio) in a 200μL volume were subcutaneously implanted into the right flanks of 8-week-old C57BL/6J male mice (The Jackson Laboratory). After 5 days, xenografted mice were assigned to 4 groups, eight each group, by stratified randomization based on tumor sizes (between 75 and 284 mm^3^). Mice were treated with KRIBB11 (65 mg/kg, i.p. injection daily, cat#T3652, Lot#132988, TargetMol Chemicals Inc.) with and without 2% L-leucine dissolved in drinking water stored in amber-colored bottles. KRIBB11 was freshly dissolved daily in the vehicle (final concentration:10% dimethylacetamide, 50% PEG300, 16% Captisol, and 24% sterile saline).

To formulate the KRIBB11 solution, KRIBB11 was first dissolved in dimethylacetamide, followed by addition of PEG300. Lastly, 40 or 50% Capitol dissolved in sterile saline was added. Tumor sizes were measured using a caliper by an animal technician blind to the study. To assess the compound’s toxicity, the body weight of tumor-bearing animals was also recorded. Tumor volumes were estimated using the formula [length (mm) × width^2^ (mm^2^)]/2.

### Quantification And Statistical Analysis

Statistical analyses were performed using Prism GraphPad 10.0 (GraphPad Software). The detailed statistical methods and sample sizes are provided in the figure legends. All results are expressed as mean±SD, mean±SEM, or median and IQR. The statistical significance is defined as: *p<0.05, **p<0.01; ***p<0.001; ****p<0.0001; n.s.: not significant. For *in vitro* experiments, sample size required was not determined a priori. The experiments were not randomized. For *in vivo* experiments, sample size required was determined by pilot experiments.

## Supplementary Material

Supplement 1
